# Increasing chemical coverage, accuracy, and reproducibility of the processing method for polar organic chemical integrative samplers

**DOI:** 10.1007/s00216-025-05746-x

**Published:** 2025-02-01

**Authors:** Matteo Baglietto, Henry MacKeown, Barbara Benedetti, Marina Di Carro, Emanuele Magi

**Affiliations:** Department of Chemistry and Industrial Chemistry, University of Genoa, Via Dodecaneso 31, 16146 Genoa, Italy

**Keywords:** Sorbent phase transfer, POCIS, LC–MS/MS, Polar analytes, Contaminants of emerging concern

## Abstract

Despite their diffusion in research studies, passive samplers are rarely used in regulatory applications. To expand the employment of passive samplers in regulatory environmental studies, standardized procedures for processing each sampler type should be proposed and accepted, but currently, each study develops its own protocol based on previous knowledge and specific needs. In this work, six identical polar organic chemical integrative samplers in seawater were deployed to understand the importance of the sorbent transfer method prior to the elution step. A common “wet transfer” with ultra-pure water was compared to a less diffused “dry-transfer,” assessing recoveries and matrix effects of 38 target compounds of emerging concern, including polar pesticides, recreational and food-related substances, pharmaceuticals, industrial additives, and ultra-violet (UV) filters. The dry-transfer procedure generally allowed better recoveries, especially for the more polar compounds, without affecting matrix effects (which remained in the range 40–130%). Along with the recovery assessment, the analysis of the non-spiked sorbent extracts revealed traces of many of the targeted emerging contaminants, quantifying perfluorooctanoic acid, UV-filters, carbamazepine, diclofenac, and triclosan. Furthermore, other compounds were found below their limits of quantitation. Ten analytes were detected only in the extracts of the dry-transferred passive samplers, highlighting the importance of applying this protocol, especially when dealing with polar compounds. This refined processing method, therefore, permits a more standardized and reproducible strategy, at the same time enlarging the set of analytes which could be detected and quantified.

**Graphical abstract:**

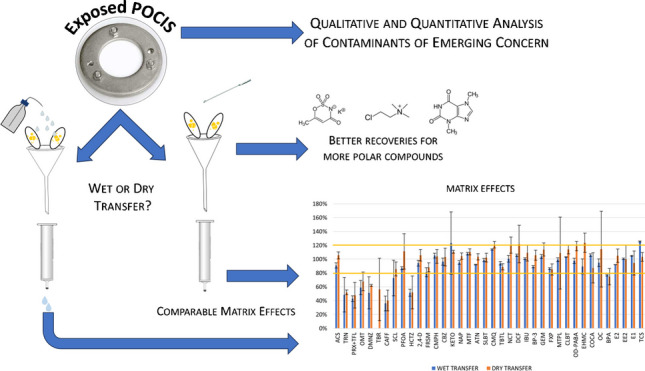

**Supplementary Information:**

The online version contains supplementary material available at 10.1007/s00216-025-05746-x.

## Introduction

The general term “emerging contaminants” (EC) refers to compounds for which there is little information about the magnitude and frequency of risks they may pose in the environment. They are not currently included in routine monitoring programs but may be candidates for future regulation. Different types of substances can be defined as EC: compounds used for decades in different applications that might have been present in the environment for many years, but whose low concentrations were detected and/or their significance (concerns) started to attract interest only recently; “true” or really “new” emerging contaminants recently introduced into the environment; well-known contaminants for which new concerns have emerged [1]. Since the beginning of the twenty-first century, a lot of scientists around the world have been focusing on this issue, and several strategies have been developed to sample, process, and detect these substances in the different natural compartments [2, 3].

To deal with the challenge of the low EC concentrations expected, devices like passive samplers were developed, allowing to greatly preconcentrate analytes of interest directly in situ. Furthermore, passive samplers are also able to combine the sampling and the purification steps. Despite their advantages, passive samplers are rarely used as a tool for regulatory environmental studies [4], also due to the lack of a standardized processing method [5]. Among passive sampling devices, polar organic chemical integrative samplers (POCIS) are the most widely diffused type for monitoring polar compounds in water matrices [3]. They consist of a sorbent placed between two protective membranes held together by stainless-steel rings. Ideally, the accumulation of analytes in the POCIS sorbent involves an initial kinetic phase, followed by a pseudolinear phase and a final equilibrium partitioning phase [6]. Thanks to the presence of the polyethersulfone (PES) diffusion-limiting membranes, POCIS are typically employed in the kinetic sampling mode as time-integrative samplers: contaminant uptake should remain linear over the whole duration of the deployment. As such, time-weighted average (TWA) concentrations can be estimated, using for each compound the mass sampled by the POCIS sorbent, the sampler exposure time, and the sampling rate (Rs), which corresponds to the volume of water from which the analyte is “cleared” by the POCIS per unit of exposure time [7]. POCIS can be deployed for a few days to some months, but the most common exposure times are 2-3 weeks.

While particular attention has been placed on studying POCIS calibration to improve its accuracy [8], much less has been done to evaluate the recovery of the processing protocol, as well as improving it, in particular the transfer step. Generally, water is employed to wash the sorbent into a solid-phase extraction (SPE)–type cartridge, but sometimes the exact transfer method is not reported in articles [9–11]. Often the volume of water employed is not specified [12–18] or only roughly reported [19, 20]. This may be problematic in terms of analyte recovery reproducibility as some of the more polar compounds may be washed away. When reported, volumes between 10 and 50 mL of water are generally employed [21–26]. In a few studies, methanol has been employed instead of water [10, 27] and in some cases the methanol wash was combined to the sorbent extract, so that the analyte fraction washed away is recovered [28–31]. Still, this implies a less green sample preparation due to the larger volume of solvent to evaporate. Additionally, it could lead to much stronger matrix effects, as all potential interfering species present are also recovered from the wash. This can be especially problematic for POCIS deployed in a marine environment due to massive presence of salts. In addition, when the wash is combined to the sorbent eluate, a fraction of the analytes of interest—especially if more hydrophobic—may originate from the membrane and not the sorbent, making the interpretation of the results more difficult. Lastly, transfer without using water nor methanol has been applied to either moist [32] or dry [33–35] sorbent. It is expected that this would also potentially lead to strong matrix effects if no further wash is carried out. So far, only Martínez Bueno et al. performed a dry transfer followed by the use of a standardized volume (2 mL) of water for further washing the sorbent in the cartridge [36].

While in a previous work of ours the elution step was studied in detail (to maximize analysis accuracy especially of mid-polar analytes) [37], the sorbent transfer from the POCIS to a glass cartridge was not optimized. Thus, the current work focused on optimizing the sorbent transfer method of POCIS deployed in seawater. Two procedures were compared using real samples, and the fractions in which the analytes partitioned during the processing were analyzed.

If the targeted contaminants are present in real samples, they are supposed to be at trace and ultra-trace levels, thus requiring an extremely sensitive and specific instrumentation. Therefore, high-performance liquid chromatography was coupled with tandem mass spectrometry (MS/MS) to maximize the chance of detecting the analytes in the POCIS extracts. According to the polarity of the analytes involved in the study, both hydrophilic liquid interaction chromatography (HILIC) and reversed phase liquid chromatography (RPLC) can be employed.

## Materials and methods

### Standards and solvents

The analytical standards used in this study were purchased from different suppliers: acesulfame (ACS), taurine (TRN), paraxanthine (PRX), theophylline (TFL), omethoate (OMT), daminozide (DMNZ), theobromine (TBR), sucralose (SCL), hydrochlorothiazide (HCTZ), chloramphenicol (CMPH), perfluorooctanoic acid (PFOA), furosemide (FRSM), 2,4-dichlorophenoxyacetic acid (2,4-D), carbamazepine (CBZ), metformin (MTF), atenolol (ATN), chlormequat (CMQ), terbutaline (TBTL), mepiquat (MPQ), nicotine (NCT), metoprolol (MTPL), clenbuterol (CLBT), ibuprofen (IBU), benzophenone-3 (BP-3), gemfibrozil (GEM), cocaine (COCA), octyldimethyl *p*-aminobenzoate (OD-PABA), ethylhexyl methoxycinnamate (EHMC), ethylexylsalicylate (EHS), octocrylene (OC), bisphenol A (BPA), estrone (E1), β-estradiol (E2), 17α-ethinyl estradiol (EE2), and triclosan (TCS) were from Sigma-Aldrich (St. Louis, MO, USA); caffeine (CAFF), ketoprofen (KET), naproxen (NAPR), and diclofenac (DCF) from Fluka Analytical (Saint Gallen, Switzerland), while salbutamol (SLBT) from Alfa Aesar (Haverhill, MA, USA). All analytical standards were equal or above 98% purity. Stock standard solutions of the 38 analytes were prepared dissolving pure standards in methanol (MeOH) or MeOH:water 1:1, and were stored in a freezer at − 20 °C.

A solution mix at 2.5 mg L^−1^ was obtained from stock solutions and employed to perform the analytes’ spikes and to prepare neat standard solutions, freshly prepared for each day of analysis.

Acetonitrile (ACN), methanol (MeOH), dichloromethane (DCM), and isopropyl alcohol (IPA) were purchased from VWR (Fontenay-sous-Bois, France), while ultra-pure (mQ) water was obtained in the lab using a Milli-Q Millipore (Watford, UK) system. Acetic acid (AA), formic acid (FA), and ammonium formate (FNH_4_) were used as chromatographic additives and provided by VWR, all of them LC–MS grade. Two salts were also necessary to carry out this study: sodium chloride (purity ≥ 99%) from Sigma-Aldrich and magnesium sulfate (99%) from Carlo Erba Reagents (Rodano, MI, Italy).

### Sample processing

The analytical development performed in this work can be summarized as follows:

- Comparison of two procedures of sorbent transfer from deployed POCIS, by spiking the analytes;
- Verification of the representativeness of the spike through a model experiment;
- Evaluation of the mass balances among the fractions involved in the processing.

The final procedure involved the transfer of the sorbent (dried overnight under a laminar fume hood) into a fritted glass cartridge with the aid of a spatula. Then, the sorbent was washed with 5 mL of mQ water before elution with 20 mL of MeOH and 5 mL of DCM:IPA, 8:2 (v/v). The extracts were evaporated to dryness (Rotavapor® R-100, BUCHI, Switzerland), reconstituted in 1 mL of methanol and filtered through a 0.2-μm hydrophilic PTFE filter, prior to dilution and final analysis through RPLC-MS/MS with the previously optimized protocol [37], whose applicability was widened by adding new compounds to monitor.

#### Polar organic chemical integrative samplers deployed in the Ligurian Sea

**Fig. 1:**
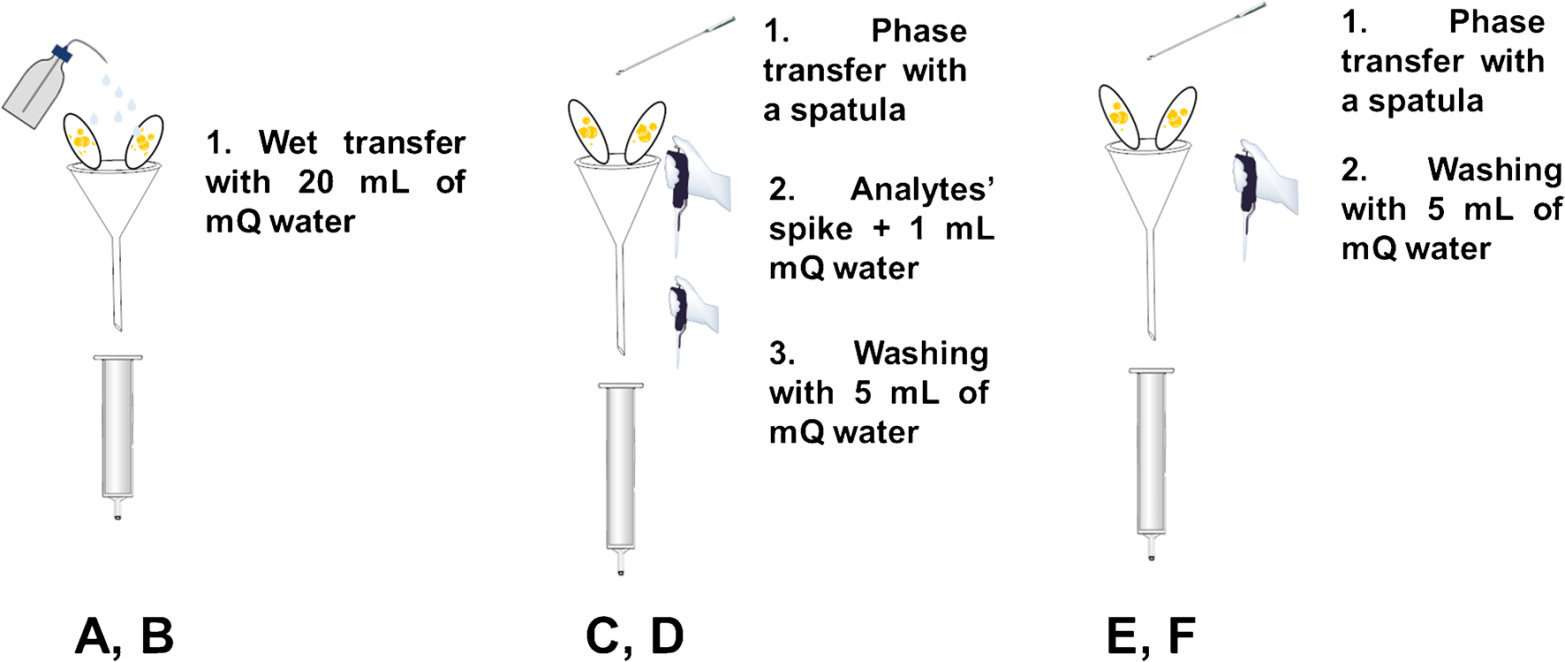
Schematic representation of the HLB sorbent phase transfer from the PES membranes to the SPE cartridges. POCIS A and B sorbents were wet-transferred with mQ water, while POCIS C–F were dry-transferred with a spatula. C and D sorbents were spiked with the analytes before washing and elution

Six commercial POCIS bought from E&H services (Prague, Czech Republic) were deployed for 12 days during September 2021 at a site on the Ligurian coast, within the protected marine area of Portofino (Genoa, Italy). An exposure time of 3 weeks was planned, but a big sea storm was forecast during the deployment and the samplers were thus retrieved in advance. After their retrieval, they were stored frozen at − 18 °C until processing. The sorbents of the six POCIS were transferred in different ways, as shown in Fig. 1.

**Fig. 2:**
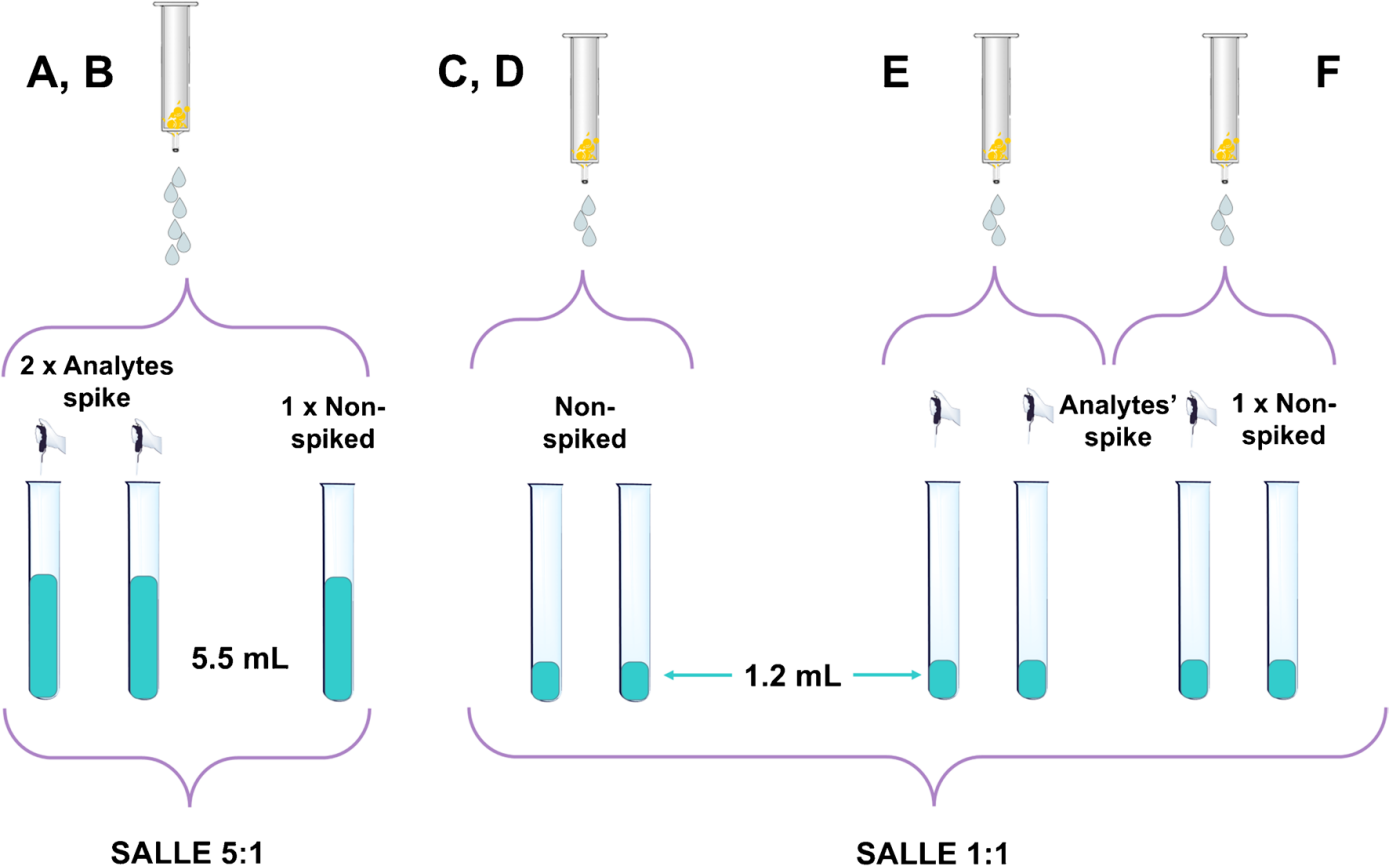
Processing of the wash waters employed during the phase transfer of the six POCIS

The first couple (POCIS “A” and “B”) was processed through a usual wet transfer [37], using 20 mL of mQ water. This water was collected and three aliquots of 5.5 mL each were further processed as follows, according to Fig. 2: two of them were spiked with 44 μL of a standard mix solution at 2.5 mg L^−1^ (to get a concentration of 20 ng mL^−1^), while the third was used as “non-spiked” aliquot (to check for the presence of the target compounds in the sample). These aliquots were subjected to a salt-assisted liquid–liquid extraction (SALLE): 1.1 mL of ACN was added (5:1, sample to extraction solvent volume ratio) to obtain a single-phase solution, which was homogenized through agitation and vortexed (VM3 vortex from CAT—Staufen, Germany) for 1 min at 2000 rpm; then, by the addition of 1.1 g of NaCl and 4.2 g of MgSO_4_, the increase in ionic strength caused phase separation, which was completed by centrifugation for 6 min at 3500 rpm (using an ALC centrifugette 4206 from Aiken Corporation – Aiken, SC, USA). The organic layer (supernatant) was finally withdrawn, filtered on 0.22 μm hydrophilic-polytetrafluoroethylene (PTFE) filters (Phenomenex—Castel Maggiore, Italy), diluted 1:10 in ACN:mQ, 95:5 (v/v) and analyzed through the HILIC-MS/MS method described below [38].

The HLB sorbent of POCIS C-F was transferred in a dry manner: POCIS were left to dry overnight under a fume hood and then the sorbent was transferred into glass cartridges with the aid of a spatula and a funnel, without using any liquid. The sorbent transferred from POCIS C and D was spiked with 200 μL of the standard mix solution at 2.5 mg L^−1^ and then each cartridge was wet with 1 mL of mQ water, prior to being stored frozen at − 18 °C overnight. The following day, they were allowed to return to room temperature and the sorbent was washed with 5 mL of mQ water, which was collected, divided in aliquots, and submitted to SALLE: two replicates of 1.2 mL for each of the waters from POCIS C and D (4 samples overall, as reported in Fig. 2) were processed with the SALLE procedure using a 1:1 ACN to mQ volume ratio [38]. A similar processing was applied to POCIS E and F, without the spike prior to the washing (see Fig. 1). Three of the four 1.2-mL aliquots of wash-waters obtained by these samplers were spiked with 48 μL of the standard mix solution at 2.5 mg L^−1^, while the fourth was used to assess the possible natural presence of the analytes in the samples’ wash (Fig. 2). The following SALLE processing was the same as for POCIS C and D. SALLE extracts were then analyzed through a HILIC-MS/MS method previously optimized [38].

On the other hand, the sorbent of each POCIS was dried and eluted with 20 mL of MeOH and 5 mL of a DCM:IPA 8:2 (v/v) mixture. Then, the extracts were processed as described above.

#### Model samples—solid phase extraction of spiked waters

The procedure described in section “Polar organic chemical integrative samplers deployed in the Ligurian Sea” was used to evaluate the recovery of the analytes from a real exposed POCIS, by spiking the sorbent after deployment. Therefore, the recovery of some analytes could be affected by salts and other matrix components that could have sorbed onto the HLB phase. To verify the interaction of all the analytes with the HLB phase in general and to obtain further information about the mass balance of the analytes in the various fractions, a simple SPE procedure was performed on spiked water in triplicate, along with a procedural blank. In detail, 6-mL glass cartridges were manually packed with 200 mg of HLB sorbent (by Waters, Vimodrone, Italy) between two polyethylene frits (Thermo Fisher Scientific, Monza, Italy). They were conditioned with 5 mL of MeOH and 5 mL of mQ water. After that, three cartridges were each loaded with 100 mL of water spiked with 40 μL of a standard mix solution at 2.5 mg L^−1^ (for a final concentration of 1 ng mL^−1^). A fourth cartridge was loaded with 100 mL of water with no spike. Each loaded sample was collected separately after passing through the SPE cartridge, diluted 1:1 with MeOH and analyzed by LC–MS/MS, to estimate the “flow-through” fraction.

Each cartridge was then washed with 5 mL of mQ water, which was collected and analyzed as well, to calculate the “washed” fraction. Afterwards, they were dried and eluted with the same protocol employed for the POCIS [37], enabling the determination of the “eluted” fraction. Finally, by knowing the spiked (total) amount of each analyte present in the water, the “non-eluted/retained” fraction could be obtained by the difference. A detailed explanation of the calculation is presented in another section (“Mass balances—experiment on the model SPE”).

### LC–MS/MS analysis

The chromatographic separations were achieved on a 1200 series HPLC coupled to a 6430 triple quadrupole mass spectrometer by Agilent Technologies (Santa Clara, CA, USA), which employed electrospray ionization (ESI) as an ion source.

The two separation strategies employed for the analyses relied on previously optimized gradient elutions: for the RPLC-MS/MS analysis of the POCIS and the SPE model experiment’s extracts, two different separations were carried out on a Kinetex® C18 Polar (100 mm × 2.1 mm; 2.6 μm particle size) by Phenomenex (Torrance, CA, USA) [39]. On the other hand, the organic extracts obtained by applying the SALLE to the wash waters were eluted in HILIC mode, on a YMC-Triart Diol-HILIC column (100 × 2.1 mm; 3 μm particle size) by YMC Co. (Kyoto, Japan) [38].

Two RPLC separations were performed, one with acidic and the other with neutral phases. The ESI source worked in “polarity switching” and negative mode, in turn, according to the preferred ionization mechanism of each analyte. In the first case, the gradient elution was with H_2_O and ACN as phase A and phase B, respectively, both containing 0.001% of AA. The elution program started with 60% A flowing at 0.3 mL min^−1^, reaching a 30% A at 0.4 mL min^−1^ at the 9th min of analysis, followed by a return to the initial conditions at min 10 (held for another 3 min), followed by 5 more minutes as post-run time. In the second method, neutral H_2_O and ACN were used (phase A and phase B, respectively) ensuring the separation and detection of TCS, BPA, and estrogens in 7 min, with a constant flow rate of 0.3 mL min^−1^ by passing from the initial conditions of 60% of phase A to 10% at min 5 which was held for a minute, before restoring the column at the initial solvent ratio at min 8.5. The column was re-equilibrated for a further 5 min. These two elution programs were run through the column compartment held at 40 °C. For an extensive description of these methods, refer to MacKeown et al. [39].

For the HILIC separation, a method considering some of the polar compounds was optimized in a previous paper of ours through a two-step design of experiments [38] and the plethora of analytes was further widened in another work [40], considering all the polar compounds monitored in this study. For separation of the polar analytes expected to be partially lost during the washing step, ultra-pure water with 0.01% FA and 0.2 mM FNH_4_ and ACN:H_2_O 95:5 with the same amount of modifiers were used as eluents A and B, respectively. Setting the column temperature at 25 °C, a combined eluent and flow gradient started from 100% at 0.1 mL min^−1^, after a step keeping the same composition at a higher flow (0.3 mL min^−1^), the strongest elution power was obtained with 36.8% of phase A, at a flow of 2.3 mL min^−1^. This separation lasted a total of 25 min, including the restoration of the initial conditions. For a complete description of the separation conditions and their optimization, refer to Baglietto et al. [40].

### Method performances

#### Experiment of POCIS deployed in the Ligurian Sea

The main aim of this work was to establish the process efficiency (recovery and matrix effect) for each analyte in the new proposed dry-transfer protocol. Hence, the fraction of analytes lost through the washing (and/or transfer) of the HLB sorbent from the POCIS to the glass cartridge needed to be assessed and compared to that in the previously employed wet-transfer procedure. Therefore, different parameters were evaluated:

1. the recovery from the wash water through the SALLE procedure (***R***_**SALLE**_)—assessed by spiking the water **after** the wash of the sorbent (POCIS E and F);
2. the fraction lost from the POCIS sorbent (and thus recovered) by the wash waters (***R***_**WASH**_)—evaluated through the analysis of the waters of POCIS C and D, whose sorbents were spiked before the washing;
3. the fraction actually eluted by the solvents (***R***_**ELUT**_)—obtained through the analysis of the eluates.

SALLE recoveries (***R***_**SALLE**_) were assessed by using the following equation:

1 $$\begin{array}{c}{R}_{\text{SALLE}}\%=\frac{{A}_{\text{B}-\text{SALLE}}-{A}_{\text{NS}}}{{A}_{\text{A}-\text{SALLE}}-{A}_{\text{NS}}}\%\end{array}$$

where ***A***_**B-SALLE**_ is the peak area obtained from the analysis of the aliquots of waters spiked before performing the SALLE (POCIS E and F), ***A***_**NS**_ is that of the non-spiked sample, while ***A***_**A-SALLE**_ is that of the sample spiked after SALLE (prior to analysis).

Recoveries of the washing step (***R***_**WASH**_) are not directly assessable, as the waters were not injected into the LC–MS/MS. Since they were submitted to the SALLE, results need to be corrected for the recovery of that step, and ***R***_**WASH**_ was then calculated as reported in Eq. 2.

2 $$\begin{array}{c}{R}_{\text{WASH}}\%=\left(\frac{{A}_{\text{B}-\text{WASH}}-{A}_{\text{NS}}}{{A}_{\text{A}-\text{WASH}}-{A}_{\text{NS}}}\right)\%/{R}_{\text{SALLE}}\end{array}$$

where ***A***_**B-WASH**_ is the peak area related to the analysis of the sample obtained washing the sorbent spiked before the washing (POCIS C and D), while ***A***_**NS**_ and ***A***_**A-WASH**_ represent the non-spiked aliquot and the one spiked after the processing, respectively.

Lastly, the recovery of the elution step (***R***_**ELUT**_) was assessed using a formula analogous to Eq. 1 but where peak areas were obtained from the POCIS extracts:

3 $$\begin{array}{c}{R}_{\text{ELUT}}\%=\frac{{A}_{\text{B}-\text{ELUT}}-{A}_{\text{NS}}}{{A}_{\text{A}-\text{ELUT}}-{A}_{\text{NS}}}\%\end{array}$$

Some guidelines recommend a reference acceptable range of 70–120% [41]. Lower recoveries are generally considered as “poor,” but values above 35% may still be used for semi-quantitative purposes, if recoveries are reproducible. The dry-transfer procedure was thus compared with the wet one in terms of absolute recoveries of the elution step, from a previous work [37]. Furthermore, the matrix effects (ME) were evaluated following Eq. 4:

4 $$\begin{array}{c}ME\%=\frac{{A}_{\text{spk}-\text{post}}-{A}_{\text{NS}}}{{A}_{\text{neat}}} \%\end{array}$$

where ***A***_**spk-post**_, ***A***_**NS**_, and ***A***_**neat**_ are the peak areas of the sample spiked post-processing (just prior to the analysis), non-spiked, and that of a neat standard (in pure solvent) containing the same amount of spiked analytes, respectively. ME is considered negligible when the alteration is lower than 20% of signal suppression/enhancement (80% < ME < 120%) and moderate if up to 50% (50% < ME < 80% ∪ 120% < ME < 150%) [41, 42].

#### Mass balances—experiment on the model SPE

To confirm these results, the model experiments carried out with spiked mQ water extracted through SPE allowed to calculate a complete mass balance:

5 $$\begin{array}{c}{M}_{\text{spiked}}={M}_{\text{f}}+{M}_{\text{w}}+{M}_{\text{e}}+{M}_{\text{n}}+e\end{array}$$

where ***M***_**spiked**_ is the mass of analyte present in the water sample; ***M***_**f**_ is the “flow-through” fraction (which passed through the cartridge without being retained by the sorbent phase); ***M***_**w**_ is the washed fraction, which interacts with the sorbent weakly/not-specifically and thus it is easily washed away; ***M***_**e**_ is the eluted fraction, the one determined in the analyzed eluate; ***M***_**n**_ is the non-eluted fraction, strongly bound to the sorbent, thus not eluted; and ***e*** represents an error, possibly due to both an under- or over-estimation of any of the other fractions.

***M***_**spiked**_ is known a priori, while the other fractions need to be calculated as shown in Eqs. 1–3, where the “non-spiked” aliquot is represented by the procedural blank, and the “spiked-after” aliquots of each step are obtained from it.

#### Instrumental-related performances

In addition to the extraction efficiency (including both recovery and matrix effect), also the instrumental sensitivity to the different substances affects the method’s limits of detection (LOD) and quantitation (LOQ). From a practical point of view, there are several strategies to calculate LODs and LOQs, and the most conservative procedure should be employed [43]. A previous study of ours employing the same instrument compared two of these strategies [38], and the selected method was the one employing the following proportion:

6 $$\begin{array}{c}(n \cdot {S}_{\text{BkM}}) :{C}_{\text{LOD}/\text{LOQ}} = {S}_{\text{SpkM}} : {C}_{\text{SpkM}}\end{array}$$

where ***n*** is 3 for LOD and 10 for LOQ, ***S***_**BkM**_ and ***S***_**SpkM**_ are the average signals (peak areas) of a blank matrix and of a spiked one, respectively, while ***C***_**LOD/LOQ**_ and ***C***_**SpkM**_ are the corresponding concentrations in the diluted extracts. To convert them into the corresponding values within the original matrix, they have to be corrected for the dilution factor and for the recovery, where necessary (when lower than 70%).

## Results and discussion

### SALLE extraction on the wash water of the new protocol

In order to check the fraction of analytes lost in the washing step, the wash waters were analyzed. An extraction/purification procedure prior to the analysis was necessary to avoid significant matrix effect due to the presence of salts. A SALLE previously developed for the extraction of some polar analytes from aqueous solutions was employed [38]. This procedure exploits a single-phase solution containing water and acetonitrile (which allows a theoretical infinite surface area during the extraction itself). By increasing the ionic strength (adding salts), a phase separation occurs, allowing the extraction of organic compounds within the acetonitrile layer [44].

The wash waters were expected to contain only the most polar fraction of what was initially sorbed onto the sorbent phase, since the non-polar compounds should mainly retain onto the sorbent during that washing step. Hence, only the most polar compounds were expected to be significantly lost during the washing step, and therefore, the extraction efficiency of SALLE was tested on the 20 most polar compounds among the targeted analytes involved in this study. Due to the polarity of the compounds of interest, hydrophilic interaction liquid chromatography (HILIC) was selected as separation strategy.

From the spikes performed directly onto the wash waters (POCIS E and F), it was possible to assess the recoveries and matrix effect of the SALLE procedure itself: Most of the compounds (16/20) were satisfactorily recovered (ranging 45–147%); NCT (26%) and CMQ (25%) were poorly recovered, while DMNZ and TRN were not recovered at all (Table S.1). Therefore, the potential loss during the washing step could be estimated for a total of 18 polar contaminants using the SALLE procedure.

To estimate the fractions of analytes washed away (***R***_**WASH**_), the concentrations found in SALLE extracts of the wash waters of POCIS sorbents spiked before the washing step (POCIS C and D) were corrected for the recoveries of the SALLE procedure itself. It resulted that ***R***_**WASH**_ was relevant only for ACS (41%) and CMQ (39%), and traces (< 10%) of OMT, TBR, ATN, NCT, and TBTL were observed. Still, ACS was recovered at low but acceptable levels even in the eluates (30%) and therefore, merely CMQ among the plethora of compounds monitored in this study could be detected only in the wash waters. Thus, the extension of the analysis to the “SALLE-treated” wash waters is not worthwhile for future campaigns. Indeed, we also need to bear in mind that there are no studies about the uptake kinetics of CMQ by POCIS, which could be non-linear.

### Comparison between the wet and dry procedures on real POCIS samples

**Fig. 3:**
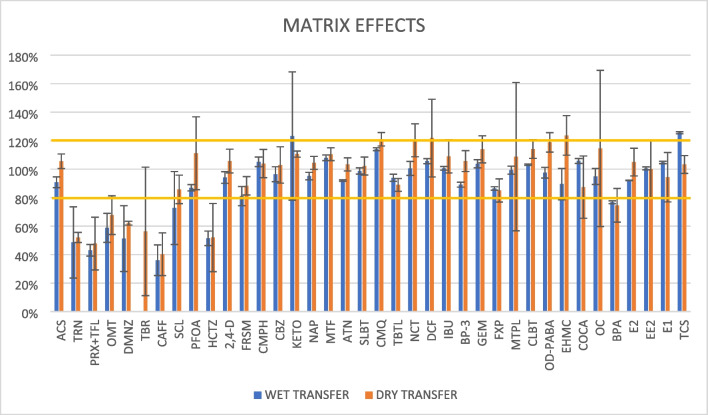
Comparison of the matrix effects obtained on extracts of POCIS co-deployed at the same time but processed with two different strategies

Table 1 reports the recoveries obtained with the two procedures. As shown, the “dry procedure” allowed to recover a larger fraction of the polar analytes than the “wet procedure.” This result was probably due to the lower volume of water used for the wash, which led to a decreased analytes’ loss in the washing step. Among the best improvements, R_ACS_ and R_SLBT_ increased by a factor of 6 and 3, respectively. The smaller volume of water used in the dry-transfer method did not negatively impact the matrix effects compared to the wet transfer method. Indeed, as shown by Fig. 3, matrix effects obtained on the diluted extracts of the two procedures were completely comparable, resulting mostly negligible (73% and 76% of the analytes for wet and dry transfer, respectively) or moderate (16% and 18% of the cases for the two strategies).

**Table 1:** Comparison of the recoveries obtained with the two procedures. *L*, low (< LOD); *NA*, not assessed

	ACS	TRN	PRX + TFL	OMT	DMNZ	TBR	SCL	CAFF	HCTZ	CMPH
R dry^b^	30 ± 11%	L	84 ± 4%	80 ± 14%	L	90 ± 4%	95 ± 19%	94 ± 15%	74 ± 3%	91 ± 11%
R wet^a^	5 ± 1%	NA	35 ± 9%*	NA	NA	NA	75 ± 8%	90 ± 14%	NA	NA
	PFOA	FRSM	2,4-D	CBZ	MTF	ATN	SLBT	CMQ	TBTL	MPQ
R dry^b^	175 ± 9%	45 ± 6%	68 ± 6%	88 ± 11%	L	70 ± 1%	59 ± 7%	L	88 ± 4%	L
R wet^a^	108 ± 3%	NA	NA	109 ± 9%	NA	NA	20 ± 2%	NA	NA	NA
	NCT	KETO	NAP	MTPL	CLBT	DCF	IBU	BP-3	GEM	COCA
R dry^b^	22 ± 3%	45 ± 15%	34 ± 11%	82.5 ± 0.4%	71 ± 3%	62 ± 3%	47 ± 5%	58 ± 5%	37 ± 4%	56 ± 1%
R wet^a^	NA	103 ± 4%	106 ± 5%	NA	NA	127 ± 12%	112 ± 7%	110 ± 5%	107 ± 6%	NA
	OD-PABA	EHMC	EHS	OC	BPA	E2	EE2	E1	TCS	
R dry^b^	89 ± 6%	91 ± 3%	93 ± 58%	91 ± 4%	138 ± 20%	103 ± 39%	98 ± 24%	102 ± 10%	113 ± 7%	
R wet^a^	58 ± 6%	97 ± 9%	NA	104 ± 9%	131 ± 21%	132 ± 34%	137 ± 27%	120 ± 16%	110 ± 6%	

^a^Values obtained from Benedetti et al. (2022) [37]

^b^Values obtained in this study

^*^Assessed as PRX only

Still, the dry-transfer method tested on deployed POCIS unexpectedly resulted in the low recoveries of some compounds, especially the anionic NSAIDs. These compounds were well recovered by the wet procedure, and simply reducing the amount of water employed to transfer/wash the sorbent phase was unlikely to have been the cause of the lower recovery. Indeed, salting out is unlikely to be the cause as the neutral polar compounds such as caffeine and sucralose were well recovered. Ion exchange on some ionic impurities of the HLB polymer is also unlikely [45], especially considering the high ionic strength of seawater, that would saturate those sites. A possible hypothesis is that in the marine environment, the POCIS sorbent could have adsorbed ions, thus creating a charged surface, able to accumulate and bind “counter-charges” at the interface [45]. In that way, the HLB sorbent exposed to seawater, after dry transfer, may have taken on the role of a mixed-mode anion/cation exchange sorbent for the spiked analytes. However, this double layer would have then been lost during the subsequent wash step, thus causing the removal of the ionic compounds adsorbed. Another possible explanation is the competition of the analytes with other species sorbed onto the phase during the deployment. As sorption is a competitive process [46], it is plausible that when spiking the “dirty” (exposed) HLB, a lower amount of the anionic analytes was able to sorb on the sorbent*.* This could be ascribed to salts which competed for ion-exchangeable sites of the HLB sorbent [47] or other species binding to the non-ionic moieties of the HLB. In general, a highly concentrated small volume of analytes was used to perform the spike. A fraction of the spiked analytes may not have been able to sorb to the salt-saturated sorbent.

These results bring once more to light the challenge in assessing recoveries from POCIS sorbent [37]. In the current study, the spike method was not adequate to correctly estimate the recoveries of the analytes that sorb to the sorbent during a field deployment. More broadly, this demonstrates that the use of internal and surrogate standards may underperform for ionic compounds in the application of POCIS to marine environments, thus leading to incorrect results.

#### Checking the hypotheses through SPE laboratory models

In order to verify the hypotheses made on the unexpected low recovery of some analytes spiked onto the exposed HLB phase, a simple SPE was performed, by loading water spiked with all analytes. By analyzing each fraction involved in the experiment (loaded water, wash water, and eluate), it was possible to calculate a mass balance for each compound, as reported in Table 2.

**Table 2:** Mass balances of the fractions studied along the recovery test in the model samples for the dry transfer procedure. Fractions: ***M***_***f***_, flow-through fraction; ***M***_***w***_, washed; ***M***_***e***_, eluted; ***M***_***n***_** + *e***, non-eluted + error (referred to any of the fractions)

Fraction	ACS	TRN	PRX + TFL	OMT	DMNZ	TBR	SCL	CAFF	HCTZ	CMPH
***M***_**f**_	97 ± 10%	166 ± 19%	T	T	99 ± 5%	T	ND	15 ± 3%	T	ND
***M***_**w**_	5.5 ± 0.1%	T	T	T	T	T	14 ± 5%	T	T	ND
***M***_**e**_	T	ND	87 ± 8%	91 ± 12%	ND	94 ± 18%	94 ± 13%	93 ± 11%	94 ± 9%	87 ± 4%
***M***_**n**_** + *e***	NS	NS	< 11%	NS	NS	NS	NS	NS	NS	< 13%
Fraction	PFOA	FRSM	2,4-D	CBZ	MTF	ATN	SLBT	CMQ	TBTL	MPQ
***M***_**f**_	T	T	T	T	93 ± 7%	T	ND	116 ± 32%	ND	130 ± 61%
***M***_**w**_	ND	ND	ND	ND	T	ND	ND	T	ND	T
***M***_**e**_	95 ± 4%	69 ± 2%	88 ± 2%	86 ± 5%	T	93 ± 4%	95 ± 4%	ND	94 ± 5%	ND
***M***_**n**_** + *e***	< 5%	< 26%	< 11%	< 14%	NS	< 7%	< 5%	NS	NS	NS
Fraction	NCT	KET	NAPR	MTPL	CLBT	DCF	IBU	BP-3	GEM	COCA
***M***_**f**_	ND	ND	T	ND	7.2 ± 0.5%	ND	ND	12 ± 1%	ND	ND
***M***_**w**_	ND	ND	ND	ND	ND	ND	ND	T	ND	ND
***M***_**e**_	43 ± 8%	87 ± 7%	85 ± 6%	81 ± 6%	83 ± 1%	81 ± 3%	86 ± 11%	86 ± 26%	84 ± 5%	83 ± 31%
***M***_**n**_** + *e***	< 57%	< 13%	< 12%	< 19%	< 10%	< 19%	< 15%	NS	< 17%	< 18%
Fraction	OD-PABA	EHMC	EHS	OC	BPA	E2	EE2	E1	TCS	
***M***_**f**_	T	17 ± 2%	ND	T	ND	ND	ND	ND	ND	
***M***_**w**_	ND	T	T	T	ND	ND	ND	ND	ND	
***M***_**e**_	25 ± 3%	30 ± 3%	23 ± 15%	23 ± 6%	89 ± 12%	87 ± 10%	93 ± 4%	88 ± 17%	99 ± 9%	
***M***_**n**_** + *e***	< 72%	< 52%	< 72%	< 72%	< 11%	< 13%	< 7%	< 12%	NS	

*T*, traces found but not quantified; *ND*, not detected; *NS*, not significant—only regarding ***M***_**n**_** + *e*** fraction, if < < 5%

**Fig. 4:**
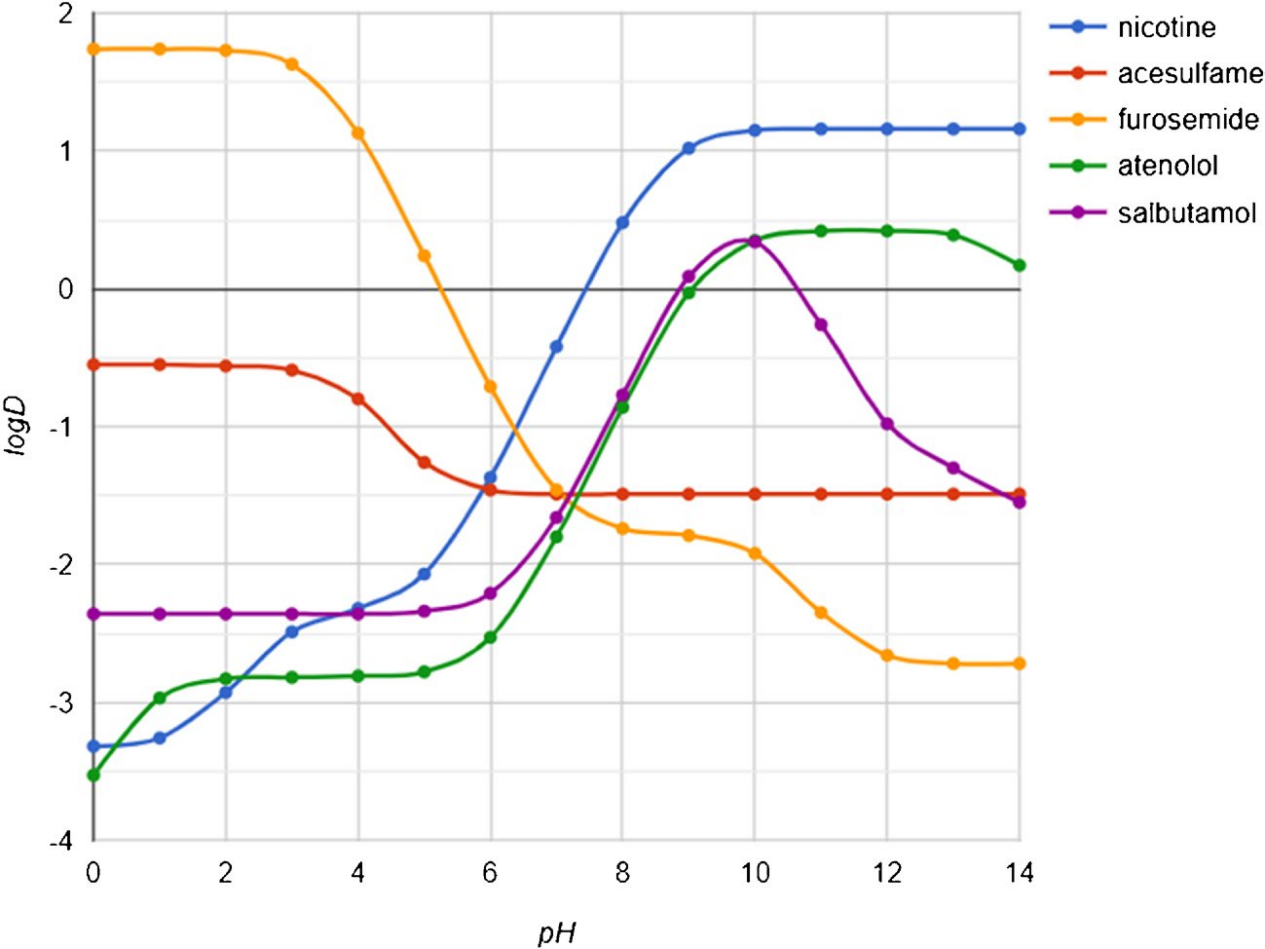
logD plots vs pH of five analytes, estimated through the logD predictor by Chemaxon (accessible at https://plugins.calculators.cxn.io/logd/).

By looking at these results, we can divide the analytes into four groups: (i) 29 compounds which behaved in an ideal manner, with recoveries of the elution fraction in the range 81–99%; (ii) the most non-polar UV-filters (OD-PABA, EHMC, EHS, OC), which resulted in poor recoveries; (iii) compounds which do not interact with the sorbent (ACS, TRN, DMNZ, MTF, CMQ, MPQ); and (iv) NCT and FRSM that behaved in an unexpected way. Group (ii) is made up of the four most non-polar compounds among the considered substances, with their logD (logarithm of the distribution coefficient, which is related to hydrophilicity/hydrophobicity, taking into account all neutral and charged forms of ionizable molecules and depending on the pH of the solution) [48] ranging from 5.11 to 6.78. In a previous work [37], we already demonstrated that the elution protocol was adequate to quantitatively elute the amount accumulated onto the sorbent. Therefore, the missing fraction (> 50% of the initial amount) may have not even reached the HLB sorbent itself but instead remained on the walls of the glass beaker because of incomplete dissolution due to their low hydro-solubility. This issue is unlikely to occur in real samples, where expected water concentrations are far lower. Interestingly, group (iii) analytes are the five most polar compounds at pH = 7 (logD ranging from − 2.62 to − 5.69) plus ACS (logD = − 1.49), which at this pH is slightly less polar than ATN and SLBT. Still, by plotting the predicted logD vs pH values (as reported in Fig. 4), it appears that at slightly basic pH (pH just above 7) ACS keeps its logD nearly constant, while both ATN’s and SLBT’s logD values increase steeply. Even the results of the group (iv) could be rationalized considering these plots: in fact, FRSM’s logD sharply decreases with increasing pH (it becomes more polar than ACS at pH > 7.0), and the fact that the ***M***_**f**_ fraction resulted only in non-quantifiable traces may be ascribed mainly to its low sensitivity, which lead to a definitely over-estimated ***M***_**n**_** + *e*** fraction; NCT presents a plot similar to that of ATN, but shifted left. Its low recovery may be due to other factors, like the fact that the free base (non-polar) is less soluble in water and more volatile than the protonated form (salts) [49]. If the pH was acid, a significant fraction of NCT could have been revealed in the flow-through and washed fractions. Therefore, the pH was probably slightly basic, and the fraction lost may be due to other phenomena, including free base NCT volatilization.

By looking at the compounds which gave low recoveries during the test on sorbent from deployed POCIS, it can be noted that during this experiment they were all well-recovered: NSAIDs ranging 81–87%, PFOA being 95%, and some cationic drugs (which suffered less of this issue) ranging 81–95%. This confirmed that the poor recovery in the previous test was due to spiking the analytes directly onto the exposed sorbent, raising the necessity to thoroughly assess the representativity of spikes during lab-studies, for example of internal standards, when dealing with solid matrices.

#### Method performances

In-matrix LODs and LOQs were calculated as described in the “Materials and methods” section and are reported in Table 3. They show a rather wide range, depending on both the extraction efficiencies and the instrumental sensitivity: for example, ACS is one of the most poorly recovered compounds, but its limits are quite low due to a good MS sensitivity for this compound; NCT was neither quantitatively recovered, nor presented good instrumental sensitivity, and therefore its limits are quite high; some of the highest LODs, such as those of TBR, EHS, and EE2, are mainly due to low MS sensitivity. In particular, the high LODs and LOQs for estrogens are due to the absence of additives in the mobile phases (avoided to keep a satisfactory chromatographic separation), such as ammonia which can be used to enhance their ionization [50].

**Table 3:** Procedural limits of detection (LOD) and quantification (LOQ) expressed in ng of analyte per gram of HLB phase

Procedure	Analyte	ACS	TRN	PRX + TFL	OMT	DMNZ	TBR	CAFF	SCL	PFOA	HCTZ
Dry transfer	LOD	3	NQ	42	22	NQ	62	16	32	1	8
	LOQ	10	NQ	126	74	NQ	187	50	97	4	24
Wet transfer	LOD	22	NA	134	NA	NA	NA	18	28	2	NA
	LOQ	65	NA	401	NA	NA	NA	55	83	6	NA
Procedure	Analyte	2,4-D	FRSM	CMPH	CBZ	KETO	NAPR	MTF	ATN	SLBT	CMQ
Dry transfer	LOD	1	2	1	0.1	20	2	NQ	0.8	0.3	1
	LOQ	3	8	5	0.5	60	6	NQ	2.5	0.9	4
Wet transfer	LOD	NA	NA	NA	0.2	20	2	NA	NA	2	NA
	LOQ	NA	NA	NA	0.5	60	6	NA	NA	5	NA
Procedure	Analyte	TBTL	NCT	DCF	IBU	BP-3	GEM	FXP	MTPL	CLBT	OD-PABA
Dry transfer	LOD	0.4	97	1	4	5	0.7	41	0.6	0.6	0.7
	LOQ	1.2	290	3	11	14	2.2	123	1.8	1.8	2.1
Wet transfer	LOD	NA	NA	1	4	6	0.8	NA	NA	NA	1
	LOQ	NA	NA	4	12	17	2.5	NA	NA	NA	4
Procedure	Analyte	EHMC	EHS	COCA	OC	BPA	E2	EE2	E1	TCS	
Dry transfer	LOD	8	349	0.2	4	7	29	50	7	1	
	LOQ	24	1047	0.5	13	20	86	150	21	4	
Wet transfer	LOD	11	NA	NA	5	5	25	36	6	1	
	LOQ	34	NA	NA	16	15	74	109	19	3	

*NQ*, not quantifiable; *NA*, not available, not studied with that procedure. Ref. to Benedetti et al. (2022) [37]

The two procedures presented no relevant differences regarding LODs and LOQs of the less polar EC for which the comparison is possible. On the other hand, the dry-transfer procedure allowed to achieve remarkably lower limits of detection and quantitation for the most polar analytes (such as ACS, PRX-TFL, SLBT). A similar behavior is expected for the other polar compounds which were not studied when the wet procedure was employed. Still, it is worth noting that for the compounds poorly recovered (< 70%) the obtained LODs and LOQs are less reliable, since they also consider the correction for the recovery itself. However, they represent an estimation of the improvement in the detection of these compounds, even though their analysis would be semi-quantitative.

Good results were obtained for most of the compounds, but TRN, DMNZ, and MTF were still insufficiently recovered, and therefore, reliable in-matrix LODs and LOQs cannot be estimated (NQ in Table 3).

### Quantification of target analytes within the studied POCIS samplers

The eluates of the deployed POCIS devices which were not spiked during processing (POCIS A, B, E, and F) were analyzed through RPLC-MS/MS to evaluate the environmental contamination of the sampling site. A chromatogram of a real sample is reported in SM (Figure S.1).

In particular, 20 of the targeted analytes were detected in at least one of the four non-spiked samples, and 8 of them were determined above their quantitation limits, including industrial additives (PFOA and TCS), pharmaceuticals (CBZ and DCF), and 4 UV filters, with concentrations ranging from 0.6 to 81 ng g^−1^ of sorbent. Further details are given in Table 4. Interestingly, half of the confirmed compounds were detected only in the sorbent extracts of POCIS transferred through the dry procedure. This further highlights the improvement in the POCIS processing obtained by applying the methodology herein proposed. The data regarding absolute amounts detected in the POCIS sorbent were used to compare devices processed in two alternative ways, but they do not provide direct information about the contamination level of the seawaters sampled.

**Table 4:** Quantification data of the POCIS deployed for this study. Amounts are given as average ± standard deviation of POCIS replicates processed in the same way, expressed in ng of analyte per gram of HLB sorbent

	ACS	CAFF	SCL	PFOA	2,4-D	CBZ	NAPR	SLBT	TBTL	DCF
Wet transf	ND	< LOQ	< LOQ	11 ± 2	ND	0.6 ± 0.3	ND	ND	ND	< LOQ
Dry transf	< LOQ	< LOQ	< LOQ	14 ± 2	< LOQ	1.3 ± 0.3	< LOQ	< LOQ	< LOQ	5.6 ± 0.9
	BP-3	GEM	MTPL	CLBT	OD-PABA	EHMC	EHS	COCA	OC	TCS
Wet transf	103 ± 7^a^	ND	ND	ND	6.7 ± 0.2	81 ± 12	ND	ND	< LOQ	9.5 ± 0.4^b^
Dry transf	42 ± 9	< LOQ	< LOQ	< LOQ	6.7 ± 0.6	34 ± 0.1	< LOQ	< LOQ	24 ± 1^*b*^	6 ± 3

^a^Data calculated on a POCIS replicate only (the other one was ND)

^b^Data calculated on a POCIS replicate only (the other one was < LOQ)

In order to obtain TWA concentrations, reliable ***R***_**s**_ are necessary. In a previous work of ours [39], many literature studies were considered to evaluate individual “median” ***R***_**s**_ for several compounds and these were proven to be good approximations. Among the ones quantified in this campaign, only three compounds (namely, PFOA, CBZ, and DCF) presented a selected value, and thus TWA concentrations were calculated for them, ranging from 0.04 to 1.11 ng L^−1^ and they are reported in Table 5. The remaining detected compounds were below their LOQ or they are too non-polar and no reliable sampling rates respecting the criteria presented in MacKeown et al. [39] were available.

**Table 5:** TWA concentrations (ng L^−1^) for the analytes detected and with reliable sampling rates available

	PFOA	CBZ	DCF
Wet transf	0.9 ± 0.2	0.04 ± 0.02	< LOQ
Dry transf	1.1 ± 0.1	0.07 ± 0.02	0.8 ± 0.2

**Fig. 5:**
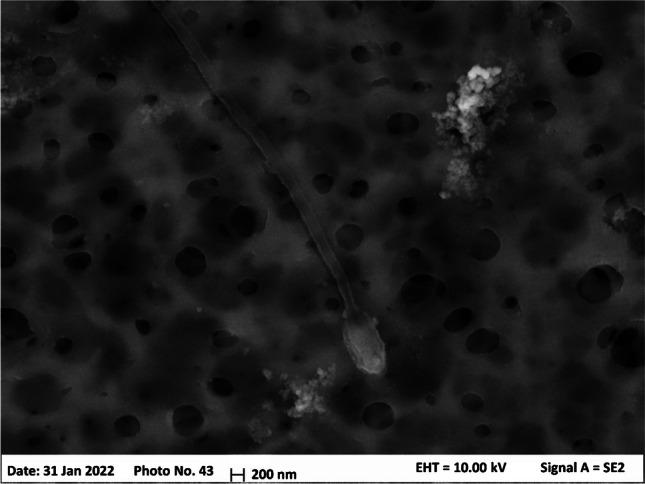
SEM image of a PES membrane after exposition of the POCIS. A filamentous microorganism is visible at the center of the image, while some spots of crystal structures (salt) are distributed along the surface

Also, TRN was actually detected in some extracts. This compound is a critical nutrient for several marine species: in fact, seafood is one of the food most abundant in TRN [51]. Despite the extremely low recovery obtained from the model experiments, relevant amounts were detected in all real samples. Of course, it is not possible to give a quantitative result since the method performances are unknown. From the literature, TRN can be found at quite high concentrations (up to some µg L^−1^) in coastal waters [52] and such high concentrations may allow significant uptake despite the low affinity with the HLB sorbent. Indeed, many microorganisms like bacteria and plankton produce TRN or they store it in their cells [53]; despite their usual dimensions (> 0.2 µm diameter), these organisms could “squeeze” through the nominal 0.1-µm PES pores [54], and reach the inner part of the POCIS. If this happens, they would be transferred and eluted along with the sorbent, leaking the taurine in their cells. The SEM image acquired onto one of the PES membranes, according to the procedure described in the SM, reported in Fig. 5, clearly shows that microorganisms (in this case a flagellate one) can stick on the membrane and partially pass through it.

This image also shows some saline crystals on the PES surface, supporting the hypothesis regarding the production of the double layer above the sorbent that may interfere with the spiked substances.

## Conclusions

The dry-transfer method for processing POCIS herein proposed presented several advantages against the more traditional wet transfer: from a practical point of view, it is simpler and operatively more reproducible; this leads to an increased number of possible analytes to be monitored. In fact, the polarity range of the compounds that can be studied was widened towards more polar compounds. Furthermore, this procedure did not cause additional problems related to matrix effect compared to the previous methodology, since the washing step resulted sufficient to remove the more interfering species (including salts). Some less satisfactory results were obtained by the experiment on exposed POCIS, but a possible explanation was proposed, then successfully tested and verified with the model SPE experiment. The improvements gained by this procedure change can be observed by looking at the increased number of analytes detected in POCIS extracts transferred with the dry protocol (20) rather than those wet-transferred (10). For three of them, a TWA estimation was also possible by using reference sampling rates.

The obtained results could help in the progression of standardization of passive samplers’ processing, towards a more reliable exploitation of this strategy.

## Supplementary Information

Below is the link to the electronic supplementary material.

Supplementary file1 (DOCX 647 KB)
