# Supplementary material for: Increasing chemical coverage, accuracy, and reproducibility of the processing method for polar organic chemical integrative samplers
Source: Anal Bioanal Chem. 2025 Feb 1;417(8):1567–80. doi: 10.1007/s00216-025-05746-x (PMC11876234; doi:10.1007/s00216-025-05746-x)
Supplement: Supplementary file 1 — Supplementary file1 (DOCX 647 KB) [file 216_2025_5746_MOESM1_ESM.docx]

Supplementary Material to the paper:

“Increasing chemical coverage, accuracy and reproducibility of the processing method for Polar Organic Chemical Integrative Samplers”

Authors: M. Baglietto, H. MacKeown, B. Benedetti, M. Di Carro, E. Magi* ([emanuele.magi@unige.it](mailto:emanuele.magi@unige.it) )

Analytical and Bioanalytical Chemistry

**Table S.1** Recoveries and matrix effects observed from wash waters of real POCIS deployed in the Ligurian Sea, following the Salt-Assisted Liquid-Liquid Extraction procedure [38] followed by HILIC-MS/MS analysis.

|  | ACS | CMPH | FRSM | HCTZ | 2,4-D | CAFF | OMT | TFL | TBR | SCL* |
| --- | --- | --- | --- | --- | --- | --- | --- | --- | --- | --- |
| REC (N=3) | 71±3% | 147±14% | 106±4% | 134±16% | 55±2% | 79±9% | 99±2% | 61±1% | 45.3±0.3% | 84±9% |
| ME | 77±15% | 119±2% | 55±7% | 54±2% | 51±0.8% | 73±10% | 56±2% | 40±2% | 40.8±0.7% | 266±19% |
|  |  |  |  |  |  |  |  |  |  |  |
|  | PRX | DMNZ | ATN | NCT | TRN | COCA | CLBT | MTPL | CMQ | TBTL |
| REC (N=3) | 57±4% | 0±0% | 46±2% | 26±3% | 0±0% | 93±2% | 113±10% | 112±6% | 25±2% | 92±7% |
| ME | 59±2% | 119±7% | 72±3% | 97±1% | 58±3% | 75.5±0.5% | 80.7±0.6% | 168±10% | 187±2% | 27±1% |

* - The values of SCL were calculated monitoring the [M+Cl]^-^ adduct. In fact, in samples with such high ionic strength, the Acetate adduct [M+CH_3_COO]^-^ usually monitored was extensively suppressed (ME<3%). The apparent enhancement is due to the lower intensity of the chlorinated adduct in neat standards injections.


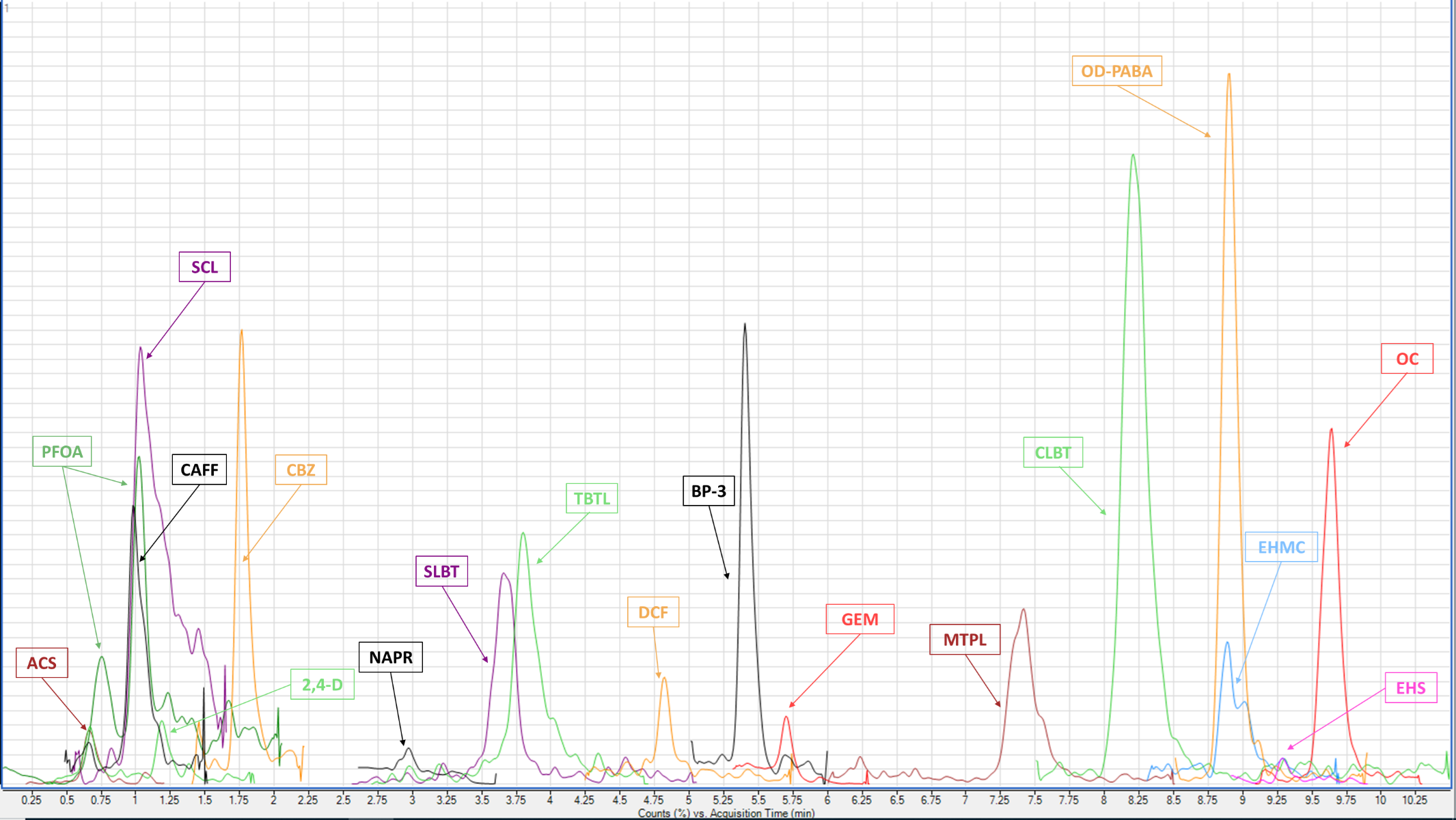
**Figure S.1** Annotated LC-MS/MS chromatogram of a real sample (POCIS F). No peak normalization was employed.

## Scanning Electron Microscope (SEM)

A small section of the PES membrane of one of the POCIS involved in this study was used to perform SEM observations. They were carried out at different magnifications (125x – 30,000x) with a Field Emission‐SEM, model ZEISS SUPRA 40 VP (White Plains, NY, USA). The sample was covered by a thin layer of graphitic carbon to render it conductive on the section to be observed.
